# Hippocampal Network Oscillations in APP/APLP2-Deficient Mice

**DOI:** 10.1371/journal.pone.0061198

**Published:** 2013-04-09

**Authors:** Xiaomin Zhang, Ulrike Herrmann, Sascha W. Weyer, Martin Both, Ulrike C. Müller, Martin Korte, Andreas Draguhn

**Affiliations:** 1 Institut für Physiologie und Pathophysiologie, Heidelberg University, Heidelberg, Germany; 2 Cellular Neurobiology, Zoological Institute, TU Braunschweig, Braunschweig, Germany; 3 Institute of Pharmacy and Molecular Biotechnology, Department of Bioinformatics and Functional Genomics, Heidelberg University, Heidelberg, Germany; University of Bristol, United Kingdom

## Abstract

The physiological function of amyloid precursor protein (APP) and its two homologues APP-like protein 1 (APLP1) and 2 (APLP2) is largely unknown. Previous work suggests that lack of APP or APLP2 impairs synaptic plasticity and spatial learning. There is, however, almost no data on the role of APP or APLP at the network level which forms a critical interface between cellular functions and behavior. We have therefore investigated memory-related synaptic and network functions in hippocampal slices from three lines of transgenic mice: APPsα-KI (mice expressing extracellular fragment of APP, corresponding to the secreted APPsα ectodomain), APLP2-KO, and combined APPsα-KI/APLP2-KO (APPsα-DM for “double mutants”). We analyzed two prominent patterns of network activity, gamma oscillations and sharp-wave ripple complexes (SPW-R). Both patterns were generally preserved in all strains. We find, however, a significantly reduced frequency of gamma oscillations in CA3 of APLP2-KO mice in comparison to APPsα-KI and WT mice. Network activity, basic synaptic transmission and short-term plasticity were unaltered in the combined mutants (APPsα-DM) which showed, however, reduced long-term potentiation (LTP). Together, our data indicate that APLP2 and the intracellular domain of APP are not essential for coherent activity patterns in the hippocampus, but have subtle effects on synaptic plasticity and fine-tuning of network oscillations.

## Introduction

Amyloid precursor protein (APP) and its two homologues APP-like protein 1 (APLP1) and 2 (APLP2) form a family of mammalian transmembrane proteins with large extracellular domains [1], [2]. APP and APLP2 are highly enriched in the brain but are also ubiquitously expressed in other tissues while APLP1 expression is restricted to neurons [1], [3]. All three proteins can be cleaved by α-, β-, and γ-secretases, but only Aß-fragments of APP produce extracellular plaques and contribute to the pathology of Alzheimer's disease [1], [4]. It has been suggested that APP may be involved in cell adhesion, neuronal maturation and synaptogenesis [5]–[7]. Overexpression of APP impairs coordinated patterns of network activity in the mouse hippocampus [8], [9]. Recent studies revealed impaired long-term potentiation (LTP) in aged APP-deficient mice [10], [11], pointing towards a role for APP in synaptic plasticity. At the behavioral level, these mice have deficits in grip strength, locomotor activity and memory formation [11]–[13]. However, there is surprisingly little information about the role of APP and APLP1/2 at the intermediate level of synaptic and neuronal networks.

While deletion of single genes from the APP/APLP family leaves mice largely intact, combined lack of APP and APLP2 results in early postnatal lethality [1], [10]. We therefore used a recently developed knockin (KI) mouse model which replaces full-length APP by its α-secretase generated, soluble extracellular APPs-alpha domain (APPsα-KI) in APLP2-deficient mice (APPsα-DM; for details see Fig. S1). While expression of this ectodomain in APPsα-KI mice rescues the phenotype of APP-deletion [11], animals carrying the combination of APPsα-KI and APLP2 deletion show deficits in spatial learning and in synaptic LTP even at young age [10]. These findings indicate that the effects of single deletions can be compensated by intact remaining homologues, while combined mutations uncover essential functions of the APP/APLP protein family.

Coherent patterns of network activity form an intermediate level between cellular and systemic functions. In the hippocampus, acquisition, consolidation and recall of spatial and declarative memories are supported by different patterns of network oscillations which entrain neuronal discharges into reproducible spatiotemporal patterns [14], [15]. These patterns form in an activity-dependent manner and have a strong link to synaptic plasticity [15], [16]. Mutants affecting synaptic plasticity may, therefore, lead to altered patterns of activity at the network level which, in turn, would result in altered learning or behavioural performance. We therefore performed a systematic comparison of slices taken from wild type (WT) mice, single mutants (APPsα-KI; APLP2-KO) and double mutants (APPsα-DM). We investigated two major types of memory-related network oscillations which can be studied *in vitro*: spontaneously occurring sharp wave-ripple complexes (SPW-R; [17]) and kainate-induced gamma oscillations [18]. Appearance of these complex patterns was similar in all genotypes, despite a slight reduction of gamma oscillation frequency in CA3 of mice lacking APLP2. Notably, network patterns of double mutants did not show any significant difference to wild-type mice. We conclude that the formation and function of hippocampal networks is largely independent from APP (intracellular domain) and APLP2.

## Materials and Methods

The generation APLP2-KO mice [19] and APPsα-KI mice has been described previously [10], [11]. We used gene targeting in ES cells to replace the endogenous APP locus with modified APP alleles and have introduced a stop codon into the APP locus behind the〈-secretase site. Thus, APPsα-KI mice express only the secreted APPsα domain under control of the endogenous promoter (Fig. S1A; [11]). Phenotypic abnormalities of full APP-KO mice (including impaired LTP) are absent in APPsα mice [11]. The mild phenotypes of APP mutants might be due to functional complementation by APLP2. We therefore generated APPsα-DM mice by crossing APPsα-KI mice onto an APLP2-deficient background. These double mutant mice have previously been characterized [10]. In brief, APP^sα/WT^/APLP2^KO/KO^ mice were inter-crossed to obtain APPsα-DM (APP^sα/sα^/APLP2^KO/KO^) animals and their APLP2-KO (APP^WT/WT^/APLP2^KO/KO^) littermate controls. The two other lines were bred by homozygous breeding of KI x KI mice or WT x WT respectively. All mouse lines were backcrossed at least 6 times to C57BL/6 mice before being used in experiments. For genotyping, the same primers were used as in [11]. All 4 strains were bred and housed under identical conditions in the same room throughout their lifetime.

Experiments on network oscillations were performed on adult (12–16 weeks) WT, APLP2-KO, APPsα-KI, and APPsα-DM mice of both sexes. The experimentalist was blind towards the genotype during recording and data analysis. All procedures were in accordance with German animal protection law and were approved by the state government of Baden-Württemberg (T–08/10). For slice preparation, mice were anesthetized with CO_2_ (60%), decapitated, and the brain was quickly transferred into cooled artificial CSF (ACSF; 1–4 °C; saturated with 95% O_2_, 5% CO_2_). Composition of ACSF was (in mM): 124 NaCl, 3.0 KCl, 1.8 MgSO_4_, 1.6 CaCl_2_, 10 Glucose, 1.25 NaH_2_PO_4_, and 26 NaHCO_3_, pH 7.4. Horizontal slices of 450 µm thickness were cut on a Vibratome (Leica VT1000 S) after trimming fontal and posterior parts of the brain. Slices were maintained at 33±0.5°C in a modified Haas-type interface chamber for at least two hours before starting recordings. Most slices for recordings were taken from the mid- to ventral portion of the hippocampus. We recorded field potentials (SPW-R and gamma oscillations) as well as unit discharges with custom-made tetrodes which were gently inserted into the pyramidal cell layers of CA3b and CA1 [20]. Electrodes were made from four twisted 12.5 µm-diameter tungsten wires (California Fine Wire). Each tetrode channel was connected to a DPA-2FX amplifier (NPI Electronics). Sharp wave-ripple complexes were recorded for 40 min after reaching a steady state following electrode insertion. Subsequently, gamma oscillations were induced by bath-application of 200 nM kainate [14], [18], [21]. Usually, gamma activity developed within 30 min under these conditions and was subsequently recorded for 40 min.

Evoked field excitatory postsynaptic potentials (fEPSPs) were recorded in acute hippocampal slices of 10–13 month old male and female WT, APLP2-KO, and APPsα-DM. Stimulation electrode was positioned in the Schaffer collateral pathway and recording was done in stratum radiatum of CA1. For baseline recordings the stimulus intensity was adjusted to 40% of the maximum slope. Baseline stimulation was set to 0.1 Hz before and after LTP inducing protocols. For LTP induction, the Theta Burst Stimulus protocol (TBS: 10 trains of 4 pulses at 100 Hz; inter-burst interval 5 Hz; 3 times) was applied [10]. Potentiation is expressed as the mean fEPSP slope relative to baseline values. Error bars indicate standard error of the mean (SEM). Investigation of basal synaptic transmission was performed by either correlating fEPSP sizes to different sizes of the fiber volley amplitudes (0.1–0.7 mV) or to defined stimulus intensities (input-output curve, 25–250 µA). Presynaptic function and short-term plasticity were explored by using paired-pulse facilitation (PPF) paradigm with inter-stimulus intervals ranging from 10 to 160 ms.

### Analysis

**Figure 1:**
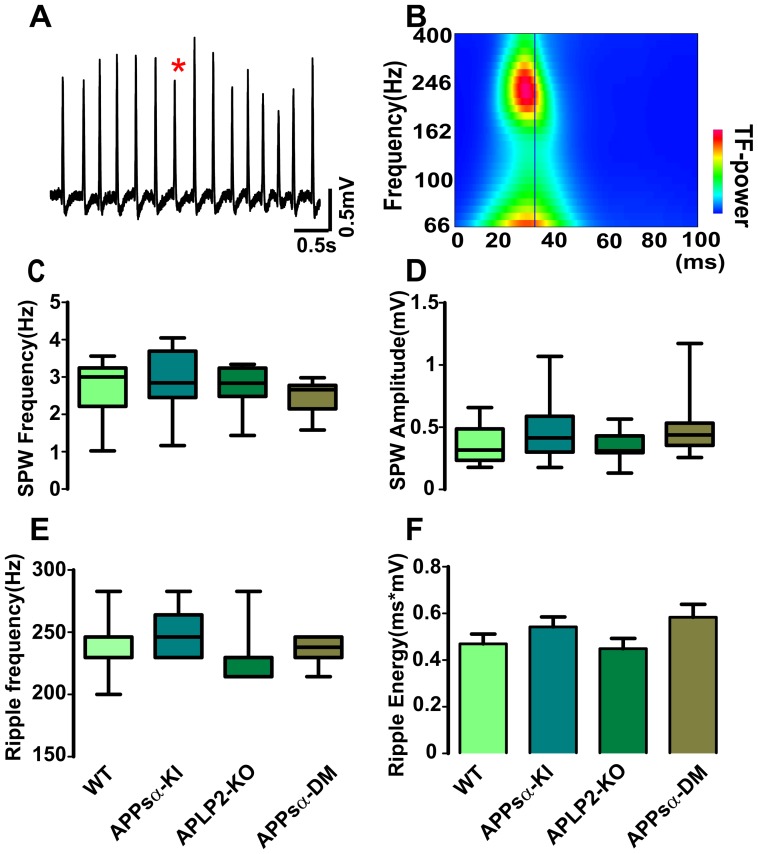
Spontaneous sharp wave-ripple complexes (SPW-R) in CA1 pyramidal layer. (A) 5 s of SPW-R raw trace recorded in the CA1 pyramidal layer. (B) Time-frequency of SPW-R, sharp waves are superimposed by high frequency ripple oscillation (∼240 Hz). (C) Medians of sharp-wave frequency are around 3 Hz in all four groups. (D) Medians of sharp-wave amplitude are around 0.4 mV, which is similar among all four groups. (E) Median of ripple frequency is slightly higher in APPsα-KI mice, in comparison to APLP2-KO mice, but without significant difference. (F) Mean of ripple energy is slightly higher in APPsα-KI and APPsα-DM mice. (n = 17/15/16/14 slices from 11/11/11/10 animals).

Gamma oscillations and SPW-R signals were amplified 100×, low-pass filtered at 10 kHz, high-pass filtered at 0.3 Hz, and digitized at 20 kHz for off-line analysis (1401 interface and Spike-2 data acquisition program; CED; Cambridge). Sharp waves were detected from low-pass filtered (<50 Hz) traces by setting a threshold at ∼4 standard deviations (SD) of event-free baseline noise [22]. Superimposed ripples were highlighted by band-pass filtering (140–320 Hz) and were accepted for analysis if their amplitudes reached more than 4 SD of event-free baseline noise. Time-frequency plots of oscillating activity were gained by continuous wavelet transform using complex Morlet wavelets, starting from 33 ms before and 67 ms after their peak [20], [22] (Fig. 1C). From this spectrogram, we extracted the leading ripple frequency and the peak power of the oscillation at frequencies higher than 140 Hz. Ripple energy was defined as the area under the spectrogram at this frequency.

**Figure 2:**
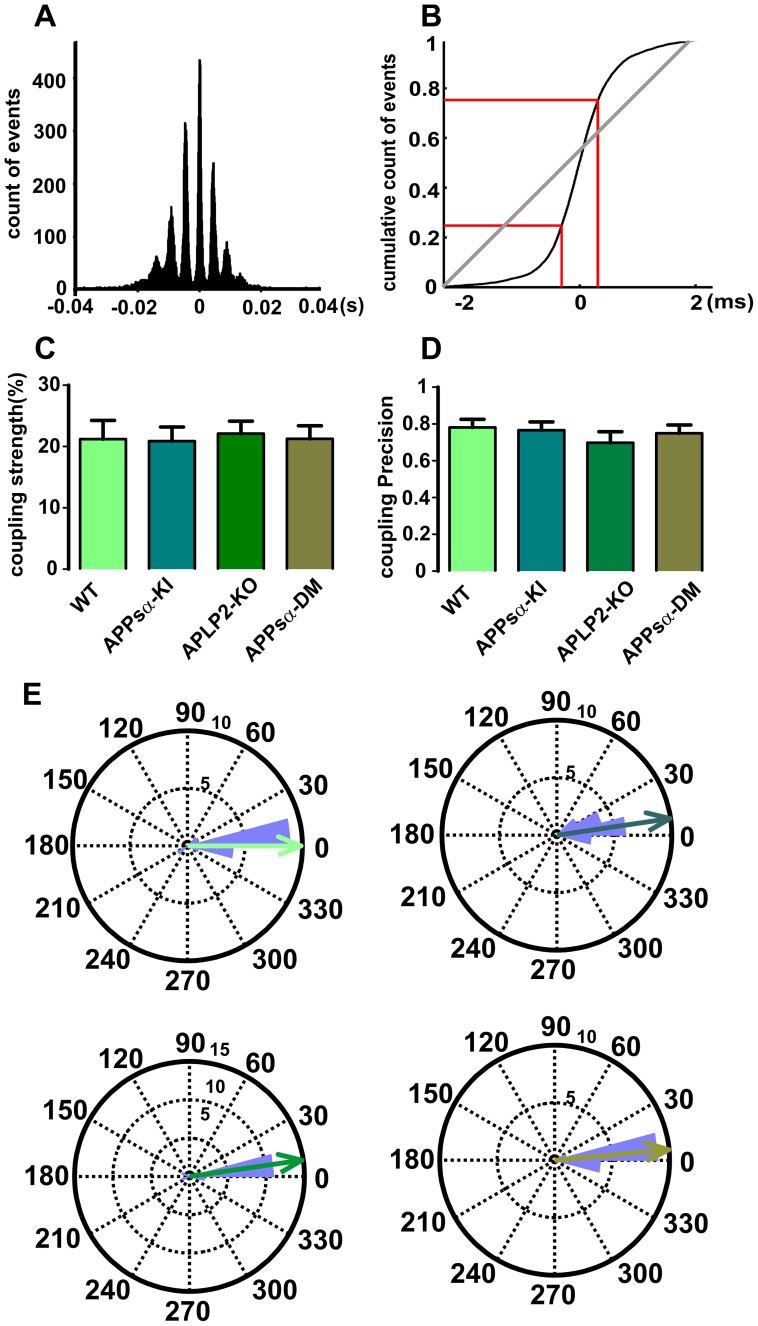
Coupling between units and ripples in CA1. (A) Cross-correlations between unit discharges in pyramidal layer and field ripples in CA1. Prominent peaks at ∼5 ms intervals reflecting the temporal coherence between action potentials and CA1 field ripples. (B) Cumulative diagram shows that more than 50% of spikes occur during less than 50% of individual ripple cycles, indicating precise temporal coupling between action potentials (‘units’) and ripples. (C) Coupling strength between units and ripples in CA1 which didn’t show any significant difference in four groups. (D) Coupling precision resemble each other among four groups. (E) Rose plots show that phase distribution of multiple units in CA1, from the slices of four different genotypes, firing phases of units to ripple troughs remained stable among different genotype.

For detection of extracellular recorded action potentials (‘units’), raw data were high-pass filtered at 500 Hz and single events were extracted by setting a threshold at four times SD to an ‘up-only’ filtered signal. To assess the firing phase of units, we set field ripple troughs to 0 and 360°, thereby defining a full cycle of the fast oscillation [23]. These data served to calculate the mean preferred firing angle and the network-entrained firing precision of units. Coupling precision quantifies the inverse of jitter of unit discharges with respect to the phase of the underlying field potential oscillation. A precision value of 1 indicates all units fire exactly at the same phase, while a precision value of 0 would mean that units diffuse firing throughout all phases. As a second measure of the temporal relationship between units and field oscillation, coupling strength was calculated from cross-correlograms of units versus ripple troughs (Fig. 2A). To quantify the coupling strength we constructed cumulative diagrams of action potentials during one full ripple cycle around the most prominent peak (maximal amplitude in the cross-correlogram (Fig. 2B)). We then calculated the fraction of the full ripple cycle in which 50% of spikes occurred. Values below 50% of the ripple cycle indicate a temporal correlation between spikes and individual ripples. This time (expressed as % of the full ripple cycle) was used as a quantitative measure of the spike ripple coupling. Lower values indicate stronger coupling [22].

For gamma oscillations, we calculated the power spectrum (Welch's method, 0.5 Hz resolution) from 180 s of gamma oscillation after 40 min of kainite perfusion, and assessed the leading frequency, amplitude, power, distinctness and coupling strength between CA3 and CA1. Mean amplitude was calculated from positive and negative peaks of raw data. Leading frequency and gamma power were defined from power spectra by an iterative process. First, we set the leading frequency to the largest power in the gamma band. We then set frequency borders for gamma at twice the half widths of the gamma peak. If necessary, leading frequency was re-adjusted such that it halved the area under the curve which was taken as the power in the gamma band. A value for the “distinctness” of gamma oscillations was calculated as the signal-to-noise ratio between peak power of gamma oscillations and the power of frequencies outside the gamma band. Coupling between CA3 and CA1 was calculated from the waveform cross-correlation which was computed in bins corresponding to the sampling rate (usually 1/20 ms) from −100 ms to +100 ms. subsequently, the largest absolute value of the cross-correlogram was taken as coupling strength and its time lag as the conduction delay.

All data sets were tested for normality of distribution with the Kolmogorov-Smirnov test. Normally distributed data were then compared by one-way ANOVA with Tukey's Multiple Comparison Test, and quantitative results are described as mean values ± SEM. If data was not normally distributed, we applied the Kruskal-Wallis test followed by Dunn's Multiple Comparison Test. Such data are described as median with confidence intervals (bottom bar indicates 2.5^th^, bottom border of box 25^th^, middle line 50^th^, upper border of box 75^th^, top bar 97.5^th^ percentile). Finally, data for all animal groups and all parameters describing network activity underwent the Holm-Bonferroni procedure to reset alpha level for multiple comparisons. Potential differences between sexes were controlled by two-way ANOVA with Bonferroni post-hoc test for normally distributed data, and Friedman test for non-normally distributed data. LTP was assessed as the ratio between averaged field-EPSP slopes from 55–60 minutes after induction and respective baseline value. Parameters from basal synaptic properties were analyzed by two-tailed student's t-test (type 2). Values of p<0.05 were regarded as significant.

## Results

Previous work shows LTP deficits in APPsα-DM mice and unaltered synaptic properties in APPsα-KI [10], [11]. Likewise, synaptic transmission and short-term plasticity are preserved in APPsα-DM (Fig. S2A-D). We then looked for alterations of coordinated hippocampal network activities in slices from single- and double-mutant mice.

Spontaneous activity was recorded at the field potential and unit level with tetrodes in CA3 and CA1 pyramidal cell layers. In the absence of any drugs, we observed regular transient increases in activity resembling SPW-R. These events emerged within CA3 and propagated through CA1 into the entorhinal cortex (Fig. 1A) [17]. Median frequency of sharp wave ripples in slices from four groups was about 2.5–3 Hz, median amplitude of sharp waves was around 0.4 mV in CA1 (Fig. 1C-D; n = 17/15/16/14 slices from 11/11/11/10 WT/APPsα-KI/APLP2-KO/APPsα-DM animals). These parameters and the basic waveform of SPW were not different between slices among different genotypes in CA1. Sharp waves were superimposed by pronounced fast field potential oscillations in the ripple frequency band between 200 and 250 Hz, consistent with previous reports (Fig. 1B; see [17], [22]). The overall frequency range of ripples in CA1 was similar for all groups despite some tendency to higher frequencies in APPsα-KI mice (∼250 Hz) as compared to APLP2-KO mice (∼230 Hz; Fig. 1E; no significant difference after applying Holm-Bonferroni test for multiple comparisons). Likewise, energy of ripple oscillations in CA1 appeared to be slightly increased in APPsα-KI and APPsα-DM mice, however, comparison with WT mice or APLP2-KO mice did not reveal a significant difference (Fig. 1F). Similar parameters were tested in recordings from CA3b pyramidal layer and did also not reveal any significant difference among the four groups (n = 13/12/13/10 slices from 11/11/11/10 mice; not different after Holm-Bonferroni test; Fig. S3A-D).

SPW-R network oscillations entrain neuronal action potentials with high precision [24]–[26]. We therefore looked whether temporal coupling between unit discharges and the surrounding field was different between any of the four genotypes. As expected, cross-correlations between unit discharges and field ripples in CA1 showed prominent peaks at ∼5 ms intervals reflecting the temporal coherence between neuronal action potentials and CA1 field ripples (Fig. 2A-B). Quantitative measures of coupling strength (see Methods) between units and field ripples in CA1 revealed similar temporal firing preferences among all four groups (Fig. 2C). Likewise, precision of temporal coupling was not different between animal lines in CA1 (Fig. 2D). Rose plots of preferred phases show that discharges occurred mostly at the trough of locally measured field ripples in all genotypes (Fig. 2E). Within CA3, coupling strength and precision appeared a bit more variable than in CA1 but did not show significant differences between genotypes (Fig. S3E-F). Again, most units fired between 0–30 degrees of ripple cycle, i.e. at the trough (Fig. S3G). Thus, our results show that discharges are similarly entrained by high-frequency oscillations in hippocampus, regardless of the absence of APLP2 or partial deletion of APP.

**Figure 3:**
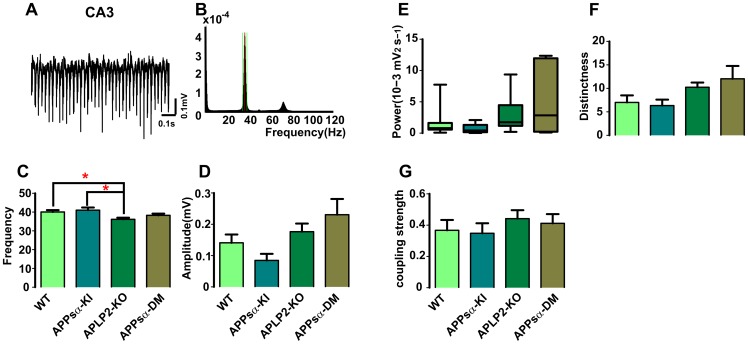
Gamma oscillations in CA3 among four groups. (A) 1 s raw traces of gamma oscillation in CA3. (B) The leading frequency and the full width of half maximum are indicated in the power spectra of CA3 (taken from the same recording as (A)). (C) Mean of gamma frequency in APLP2-KO mice is significantly decreased in comparison to WT and APPsα-KI mice. (D) Gamma amplitude is gradually enhanced from APPsα-KI, APLP2-KO to APPsα-DM in CA3, but without significant difference. (E) Gamma power shows similar trend as gamma amplitude, but doesn’t reach the significant level. (F) Gamma distinctness in CA3 is gradually enhanced. (G) Coupling strength between CA3 to CA1 is slightly weaker in APPsα-KI mice in comparison to APLP2-KO mice. (n = 8/7/9/7 slices from 7/6/7/6 animals).

We then investigated gamma oscillations, another prominent type of network activity in the hippocampus. This pattern was reliably induced in both hippocampal sub-fields within 30 min following bath-application of 200 nM kainate (Fig. 3A for CA3 and Fig. S4A for CA1). In CA3, leading frequency of gamma oscillations was significantly lower in APLP2-KO in comparison to WT and to APPsα-KI mice (n = 8/7/9/7 slices from 7/6/7/6 animals; P = 0.0096; Fig. 3C). We further quantified amplitude, power, distinctness and coupling strength between CA3 and CA1. Despite some trend towards higher gamma oscillation amplitudes in CA3 of APLP2-KO and APPsα-DM mice, this parameter was not significantly different from any other genotype (Fig. 3D). Gamma power and distinctness showed similar trends which did also not reach significance (Fig. 3E-F). Gamma coupling between CA3 to CA1 appeared slightly lower in APPsα-KI as compared to APLP2-KO mice (Fig. 3G; not significant). A similar analysis of gamma oscillations in CA1 did not show any significant differences between genotypes (n = 12/12/14/10 slices from 8/8/8/7 animals; not different after Holm-Bonferroni Test; Fig. S4A-F). In summary, gamma oscillations were only marginally affected in APLP2-KO (frequency) and normal in all other genotypes and parameters.

## Discussion

Our work focused on hippocampal network oscillations which are important for acquisition and consolidation of spatial memory [27], [28]. Despite altered synaptic plasticity in several APP/APLP2 mutants [10], [11]; coordinated network patterns have not been systematically studied with respect to these molecules. Our findings indicate that neither APLP2 nor the intracellular signaling domain of APP is essential for generation of coherent hippocampal network oscillations. We did find, however, a significantly higher gamma frequency in CA3 of APPsα-KI mice compared to APLP2-KO mice, indicating that APLP2 and APPsα play some role in hippocampal network oscillations. Furthermore, gamma frequency is significantly lower in APLP2-KO mice compared to WT mice, which may explain why there is no alteration in APPsα-DM mice. Sharp waves and ripples remained completely intact in all three genetic variants, including the very precise phase-locking of unit discharges. In summary, coordinated hippocampal network patterns are not disrupted by genetic ablation of APLP2 and the intracellular portion of APP.

In the mammalian hippocampus, SPW-R and gamma oscillation are two prominent network patterns with highly synchronized neuronal behavior [14], [26]. Although their occurrence in normal animals is state-dependent and mutually exclusive, they do share a number of common mechanisms [15], [29], [30]. A key organizing element for both network patterns is synaptic inhibition [25], [31], [32]. Combined structural and functional evidence shows that gamma oscillations and SPW-R require a precise interplay between inhibitory interneurons and excitatory pyramidal cells [17], [26], [31], [33]. Mutants affecting APP and APLP2 show altered synaptic properties of the excitatory Schaffer collateral pathway [10], [11], [34]. Interestingly, deficient LTP in APP-KO and APPsα-DM mice can be rescued by blocking GABA-A receptors, pointing towards some deficit in inhibition [10], [35]. Along the same lines, APP-KO mice have a reduced threshold for kainate-induced seizures [36]. These findings indicate that APP and APLP2 are involved in mechanisms which support the complex excitatory-inhibitory interactions needed for coherent network oscillations. We did, however, not see qualitative or major quantitative changes of the two different activity patterns studied. We conclude that excitation-inhibition-balance, intrinsic cellular excitability, and neuronal coupling are not strongly altered in any of the mutants. The mild phenotypes of all mutants may be explained by mutual compensation between different members of the APP/APLP protein family which seem to have distinct, yet overlapping functions [1], [2]. In the present experiments on network oscillations, differences were only present for APLP2-KO while partial deletion of APP did not seem to add much to the functional deficits. This is in line with previous findings showing that the soluble extracellular domain of APP alone rescues several anatomical and functional deficits of APP knockout mice [11].

In summary, our results show that full or partial deletions of proteins from the APP/APLP family cause subtle alterations at the synaptic and network level without disrupting major neuronal functions. While such data may give some hints towards the physiological functions of APP and APLP1/2, the main conclusion is that neither APLP2 nor membrane anchored full length APP have key function in organizing network behavior.

## Supporting Information

Figure S1 **APP structure and APPsα truncation generated by knockin technology.** Scheme depicting APP (APP-WT) harbouring the Aβ sequence (red) and a prominent C-terminal YENPTY protein interaction motiv. APPsα knockin (KI) mice were obtained by gene targeting in ES cells (for details see Ring et al., 2007). These mice express APPsα under control of the endogenous APP promoter. A stop codon had been introduced behind the α-secretase cleavage site into the endogenous APP locus. APPsα-KI (APP^sα/sα^APLP2^+/+^) and APLP2-KO (APP^+/+^APLP2^−/−^) mice were mated to obtain APP^sα/+^APLP2^−/−^ mice that were further intercrossed to obtain the double mutants APPsα-DM (APP^sα/sα^APLP2^−/−^) and the corresponding APLP2-KO (APP^+/+^APLP2^−/−^) littermate controls [11].
(TIF)

Figure S2 **Synaptic transmission and plasticity in three different mouse lines.** (A) LTP recording in the CA3-CA1 Schaffer collateral pathway. Single experiments are plotted showing the tendency of a LTP defect in APPsα-DM compared to control WT and APLP2-KO mice. Insets show original traces of representative individual experiments; vertical scale bar  = 1 mV, horizontal scale bar = 5 ms. (B) Input output curves of APLP2-KO and APPsα-DM mice are not different to each other (n = 11/8 slices from 4/3 animals). (C) fEPSP slope measured at defined Fiber Volley amplitudes are unaltered for APPsα-DM and the APLP2-KO mice (n = 15/14 slices from 5/5 mice). (D) Analysis of presynaptic function assessed with Paired Pulse Paradigm revealed no significant defect (n = 16/16 slices from 5/3 mice).
(TIF)

Figure S3 **Sharp-wave ripples in CA3.** (A) The mean of SPW frequency in CA3 is around 2 to 2.5 Hz in all four groups, which are not significantly different. (B) The median of SPW amplitude in CA3 is around 0.3 mV in all four groups. (C) Ripple frequency are slightly decreased in APLP2-KO and APPsα-DM mice comparing to WT and APPsα-KI mice. (D) The distribution of ripple energy in CA3 is slightly increased in APLP2-KO and APPsα-DM mice. (E) Coupling strength in CA3 is slightly higher in APPsα-KI mice, but the difference is not significant. (F) Coupling precision varies in CA3, but there are no significant differences among four groups. (G) Rose plots show that the distribution of firing phase in CA3 in all four groups is similar, with most units firing between 0–30 degrees. (n = 13/12/13/10 slices from 11/11/11/10 mice).
(TIF)

Figure S4 **Gamma oscillations in CA1.** (A) 1 s raw trace of Gamma oscillations in CA1. (B) Power spectra shows the leading frequency is around 40 Hz in CA1. (C) The mean gamma frequency is between 35–40 Hz. (D) Gamma amplitude is slightly higher in APPsα-KI mice compared to APLP2-KO mice but without significant difference. (E) Gamma power shows the similar trend as gamma amplitude. (F) Gamma distinctness is gradually enhanced from APPsα-KI, APLP2-KO to APPsα-DM mice. (n = 12/12/14/10 slices from 8/8/8/7 animals).
(TIF)
