# Supplementary figures and images for: Hippocampal Network Oscillations in APP/APLP2-Deficient Mice
Source: PLoS One. 2013 Apr 9;8(4):e61198. doi: 10.1371/journal.pone.0061198 (PMC3621758; doi:10.1371/journal.pone.0061198)

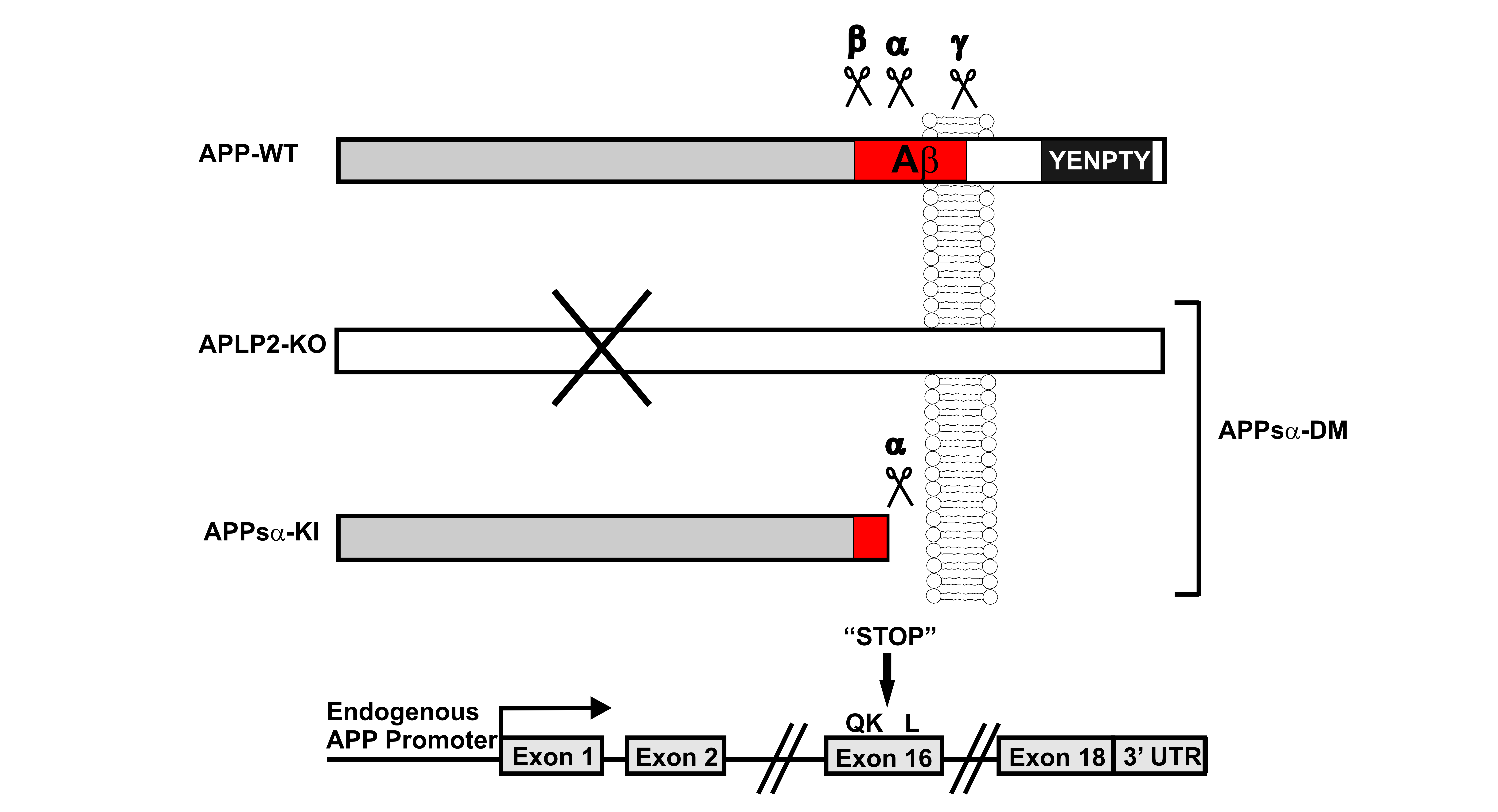

Supplement: Figure S1 — APP structure and APPsα truncation generated by knockin technology. Scheme depicting APP (APP-WT) harbouring the Aβ sequence (red) and a prominent C-terminal YENPTY protein interaction motiv. APPsα knockin (KI) mice were obtained by gene targeting in ES cells (for details see Ring et al., 2007). These mice express APPsα under control of the endogenous APP promoter. A stop codon had been introduced behind the α-secretase cleavage site into the endogenous APP locus. APPsα-KI (APPsα/sαAPLP2+/+) and APLP2-KO (APP+/+APLP2−/−) mice were mated to obtain APPsα/+APLP2−/− mice that were further intercrossed to obtain the double mutants APPsα-DM (APPsα/sαAPLP2−/−) and the corresponding APLP2-KO (APP+/+APLP2−/−) littermate controls [11]. (TIF) [file pone.0061198.s001.tif]

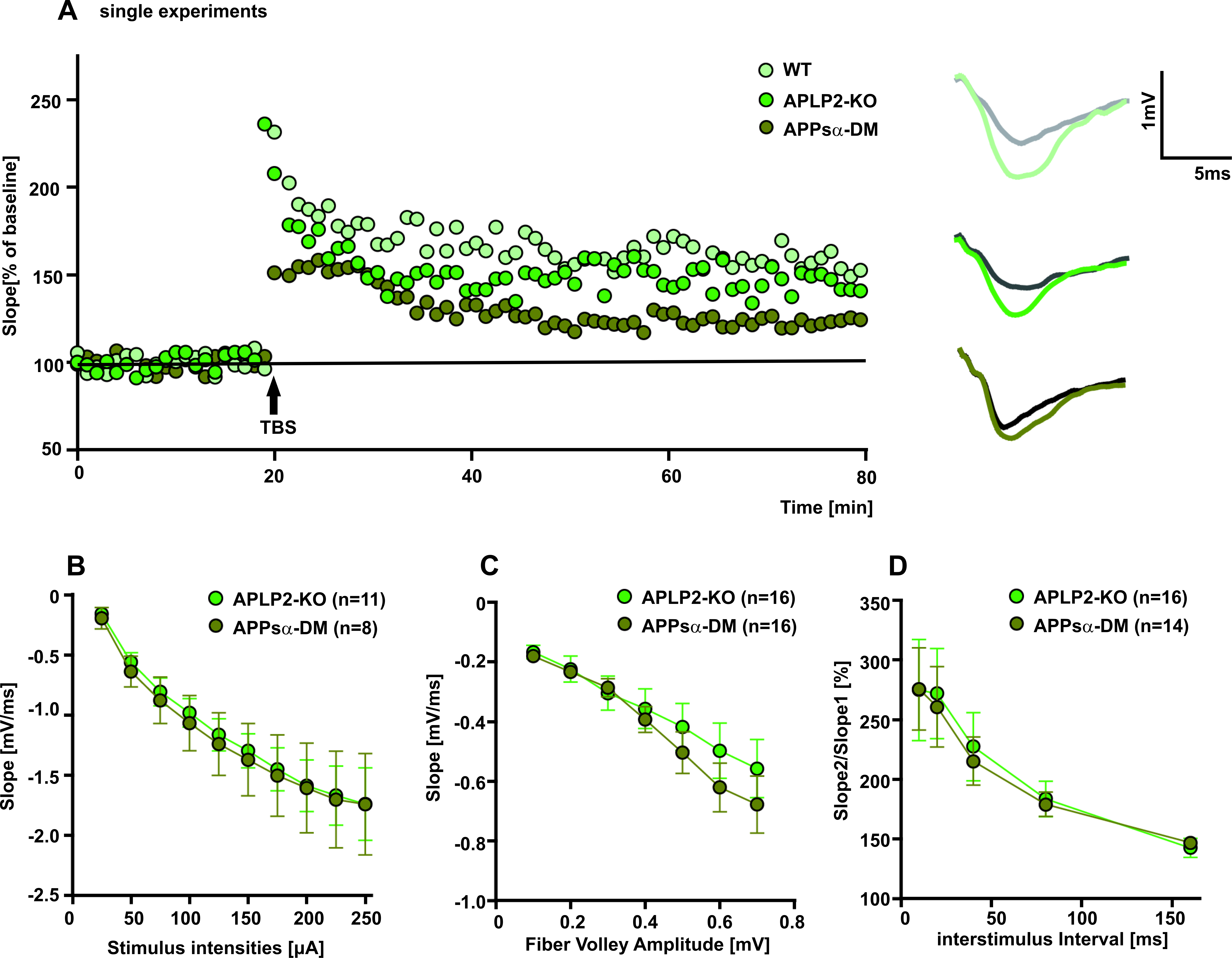

Supplement: Figure S2 — Synaptic transmission and plasticity in three different mouse lines. (A) LTP recording in the CA3-CA1 Schaffer collateral pathway. Single experiments are plotted showing the tendency of a LTP defect in APPsα-DM compared to control WT and APLP2-KO mice. Insets show original traces of representative individual experiments; vertical scale bar = 1 mV, horizontal scale bar = 5 ms. (B) Input output curves of APLP2-KO and APPsα-DM mice are not different to each other (n = 11/8 slices from 4/3 animals). (C) fEPSP slope measured at defined Fiber Volley amplitudes are unaltered for APPsα-DM and the APLP2-KO mice (n = 15/14 slices from 5/5 mice). (D) Analysis of presynaptic function assessed with Paired Pulse Paradigm revealed no significant defect (n = 16/16 slices from 5/3 mice). (TIF) [file pone.0061198.s002.tif]

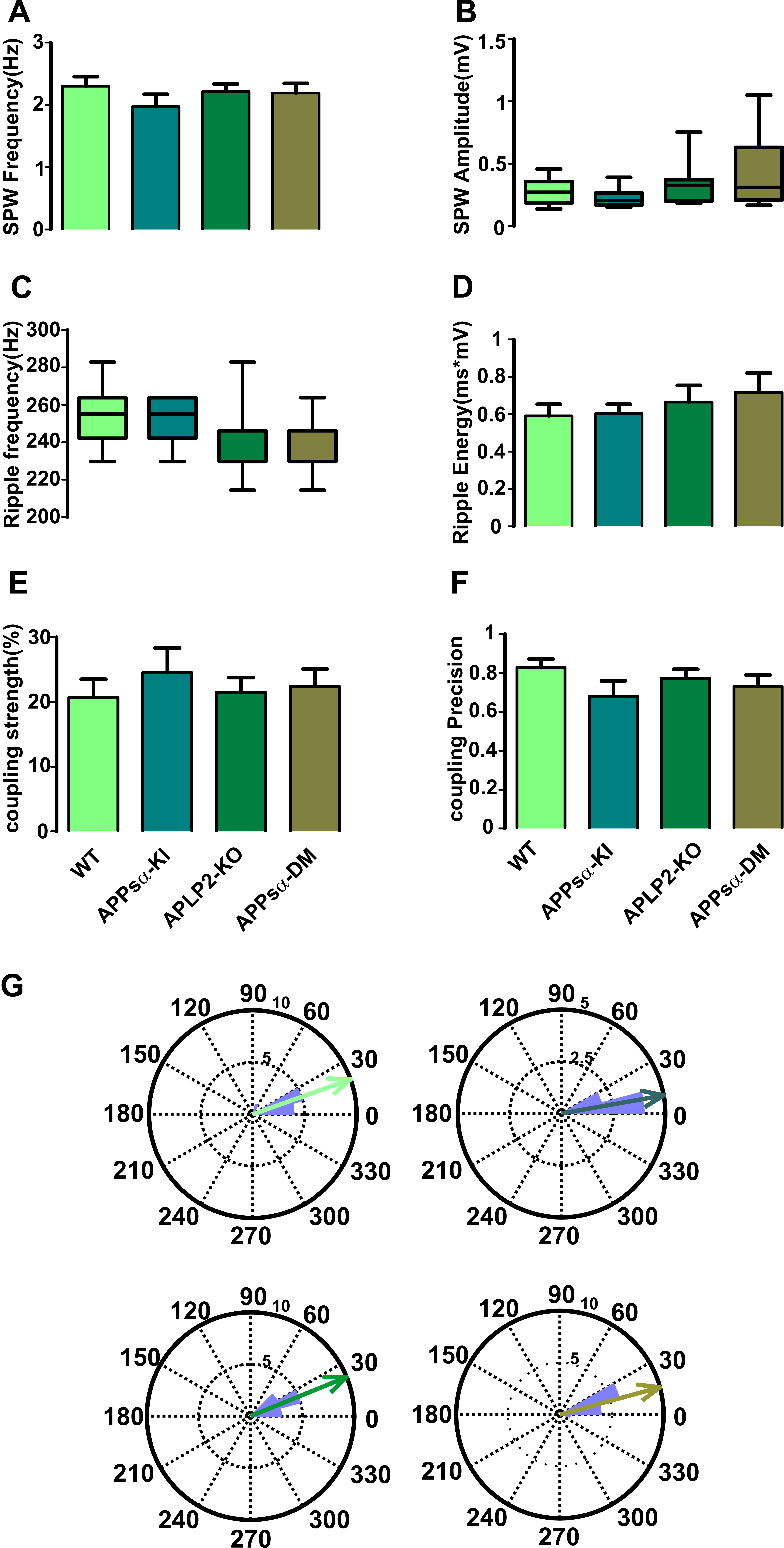

Supplement: Figure S3 — Sharp-wave ripples in CA3. (A) The mean of SPW frequency in CA3 is around 2 to 2.5 Hz in all four groups, which are not significantly different. (B) The median of SPW amplitude in CA3 is around 0.3 mV in all four groups. (C) Ripple frequency are slightly decreased in APLP2-KO and APPsα-DM mice comparing to WT and APPsα-KI mice. (D) The distribution of ripple energy in CA3 is slightly increased in APLP2-KO and APPsα-DM mice. (E) Coupling strength in CA3 is slightly higher in APPsα-KI mice, but the difference is not significant. (F) Coupling precision varies in CA3, but there are no significant differences among four groups. (G) Rose plots show that the distribution of firing phase in CA3 in all four groups is similar, with most units firing between 0–30 degrees. (n = 13/12/13/10 slices from 11/11/11/10 mice). (TIF) [file pone.0061198.s003.tif]

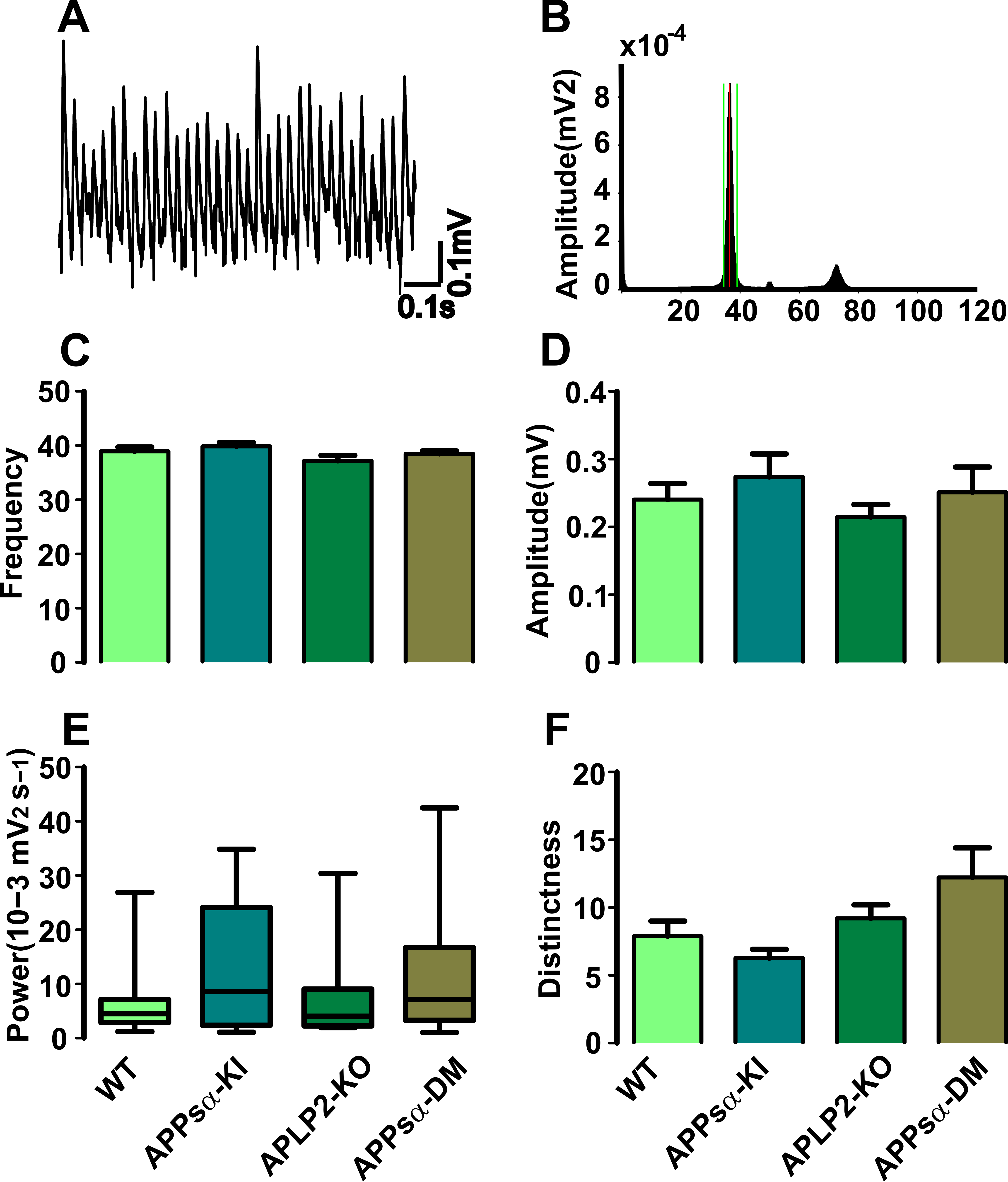

Supplement: Figure S4 — Gamma oscillations in CA1. (A) 1 s raw trace of Gamma oscillations in CA1. (B) Power spectra shows the leading frequency is around 40 Hz in CA1. (C) The mean gamma frequency is between 35–40 Hz. (D) Gamma amplitude is slightly higher in APPsα-KI mice compared to APLP2-KO mice but without significant difference. (E) Gamma power shows the similar trend as gamma amplitude. (F) Gamma distinctness is gradually enhanced from APPsα-KI, APLP2-KO to APPsα-DM mice. (n = 12/12/14/10 slices from 8/8/8/7 animals). (TIF) [file pone.0061198.s004.tif]
